# Epidemiological and clinical profile of pediatric hepatitis B virus infections in Wuhan: a retrospective cohort study

**DOI:** 10.1186/s12887-023-04460-w

**Published:** 2023-12-16

**Authors:** Jun Wang, Yong-guo Huang, Ye Zeng, Qin-zhen Cai, Mo Wu, Xin Shen, Wen-bin Tuo, Si Xie, Xiang Ma, Yun Xiang, Chun-hui Yuan, Cong Yao

**Affiliations:** 1 Department of Laboratory Medicine, Wuhan Children’s Hospital (Wuhan Maternal and Child Healthcare Hospital), Tongji Medical College, Huazhong University of Science & Technology, Wuhan, 430016 Hubei P.R. China; 2 Health Care Department, Wuhan Children’s Hospital (Wuhan Maternal and Child Healthcare Hospital), Tongji Medical College, Huazhong University of Science & Technology, Wuhan, 430016 Hubei P.R. China

**Keywords:** Children, Hepatitis B virus, Epidemiological characteristic, Serological pattern, Surveillance

## Abstract

**Background:**

Hepatitis B virus (HBV) remains a substantial public health safety concern drawing considerable attention in China and globally. The detection of HBV serological markers can enable the assessment of HBV infection and replication status in vivo and evaluate the body’s protection against HBV. Therefore, this study aims to identify the epidemiological and clinical characteristics of HBV infection in children to prevent and control HBV infection in Wuhan areas.

**Methods:**

We conducted an extensive retrospective cohort analysis of 115,029 individuals aged 0–18 years who underwent HBV serological markers detection for HBV infection in hospital between 2018 and 2021 using Electrochemiluminescence immunoassay. We generated descriptive statistics and analysed HBV infection’s epidemiological and clinical characteristics between different sex and age groups.

**Results:**

The overall positive detection rates of HBsAg, HBsAb, HBeAg, HBeAb, and HBcAb in all participants were 0.13%, 79.09%, 0.17%, 2.81%, and 5.82%, respectively. The positive rate of HBeAb and HBcAb in males was significantly lower than that in females (2.64% vs. 3.13%, 5.56% vs. 6.29%) (*P* < 0.05). Twenty-two distinct HBV serological expression patterns were revealed. Among them, 8 common expression patterns accounted for 99.63%, while the remaining 14 uncommon expression patterns were primarily observed in neonatal patients with HBV infection. There are no significant differences in serological patterns based on sex (*P* < 0.05). The overall HBV infection detection rate was 5.82% [range 5.68–5.95] and showed a declining yearly trend. The rate in females was higher than that in males 6.29% [6.05, 6.35] vs. 5.56% [5.39, 5.59]. The overall HBV diagnostic rate over 4 years was 0.20% [0.17, 0.22], and the rate declined yearly. The prevalence of acute infection was higher than that of other infection types before 2019, but the incidence of unclassified infection showed a significant upward trend after 2019.

**Conclusions:**

While the overall HBV infection detection rate in children has decreased year by year, the infection rate remains high in children under one year and between 4 and 18 years. This continued prevalence warrants heightened attention and vigilance.

**Supplementary Information:**

The online version contains supplementary material available at 10.1186/s12887-023-04460-w.

## Introduction

Hepatitis B virus (HBV) infection is a prevalent chronic viral infection worldwide and is associated with a high risk of inflammatory liver lesions leading to liver cirrhosis and hepatocellular carcinoma (HCC) [1]. Despite years of unremitting effort and the widespread availability of vaccination, HBV infection still cannot be completely cured and the infection rates remain high in some areas [2]. Therefore, reducing the infection rate, or even eliminating HBV infection, has become a major challenge to global public health security.

HBV infection occurs by horizontal or vertical transmission. Vertical transmission occurs mainly from mother to child, accounting for approximately 70–90% of infections in infants and children [3, 4]. Newborns, or children, have a natural immune tolerance which makes them often present with asymptomatic infections and develop a chronic hepatitis characterized by high viral replication, a low-inflammation phase, and normal or only slightly raised aminotransferases [5]. The disease in children is thought to have a benign course [6],drug side effects are not permitted [7] and a conservative approach is usually recommended [8, 9]. To date, the U.S. FDA and the European Medicines Agency have approved eight antiviral drugs, for children older than 1 year, to suppress viral replication and reduce disease progression to cirrhosis and HCC [4, 5]. HBV infection in children is still a huge challenge and in-depth exploration of its clinical epidemiological characteristics is important to reduce HBV infection in children.

The World Health Organization (WHO) recommends a three-dose HBV vaccine as the most effective way to prevent infection and the chronic sequelae of cirrhosis and HCC. It can prevent more than 90% of vertical transmission to infants, and more than 95% of horizontal transmission during childhood and adulthood [10]. In China, the Ministry of Health has provided free complete HBV vaccination for all new-borns since 2002 [11]. According to health statistics in 2015, since the implementation of HBV vaccination, the incidence of HBV infection in China has dropped from 8 to 15% at the peak to less than 1% in 2015 [12, 13], making an important contribution to the WHO’s goal of eliminating HBV as a public health threat by 2030 [14].Although many effective measures have been taken to prevent the spread of HBV, infants and children are still vulnerable to infection due to a variety of difficulties, such as latent HBV carriers, lack of pre-pregnancy examination of couples, prenatal infection of pregnant women, failure of prenatal virus blocking and immunization of children, lack of cooperation between obstetrics and paediatrics, and close contact within the family [10, 15]. Therefore, faced with these uncontrollable situations, it is necessary to further analyse the epidemiological situation and clinical characteristics of HBV infection.

In this study, we enrolled 115,029 individuals from our hospital and systematically analysed the prevalence and clinical characteristics of children with HBV infection in the Wuhan area, which may provide insight for assessing HBV infection status and the protective effect of the HBV vaccine and developing strategic plans for the preventing and treating HBV infection in children.

## Materials and methods

### Study population

We performed a retrospective investigation including 115,029 individuals who underwent medical evaluations as outpatient or inpatient for HBV infection detection from January 2018 to December 2021 in Wuhan Children’s Hospital. Inclusion criteria: (1) all medical examination, outpatient and inpatient children with HBV infection detection in our hospital; (2) When multiple serological tests were performed on the same enrolled subject within a year, if the test results were consistent, only one of them was taken. If the test results were inconsistent, both tests were included. (3) children under 18 years old and living in Wuhan; (4) detailed and complete demographic and clinical data of HBV-positive children. Exclusion criteria: (1) When multiple serological tests were performed on the same enrolled subject within a year, duplicate consistent results would be taken only once, and the remaining duplicate results were excluded. (2) children living outside Wuhan were excluded; (3) children with incomplete demographic and clinical data were excluded.

Routine HBV infection detections include five quantitative serological markers (HBsAg: ①, HBsAb: ②, HBeAg: ③, HBeAb: ④, and HBcAb: ⑤), and the diagnosis of HBV infection was based on the Diagnostic criteria for viral hepatitis B (WS299-2008) [16]. According to the diagnostic criteria, patients with confirmed HBV infection were divided into acute HBV patients (HBsAg changed from negative to positive within 6 months, and HBV-related symptoms or signs appeared for the first time recently; the note column of the report card was clear as acute HBV.), chronic HBV patients (HBsAg positive > 6 months; the note column of the report card was clear as chronic HBV.) and unclassified HBV patients (The information was missing or the classification could not be clearly classified for other reasons) for the following-up study. The clinical data collection included age, gender, and serological markers. All participants were divided into six groups based on age: 1–31 (D), 1–12 (M), 1–3 (Y), 4–6(Y), 7–12 (Y), and 13–18 (Y).

### HBV serological screening assay

We collected 2–4 mL venous blood from all participant and centrifuged at 3000 rpm for 6 min at 4 °C to separate the serum. The Roche cobas 8000 e602 Immunoassay Analyzer used Electrochemiluminescence immunoassay (ECLIA) to measure the HBV serological markers screening, and the detection reagents were provided by Roch (Roche Diagnostics, Germany). The analyser system diagnostics, calibration, and quality control for all assays run on it passed specifications before testing. Moreover, all samples with lipidaemia and/or haemolysis were excluded. According to Roche manufacturer’s reagent instructions, HBsAg and HBeAg antigen titer at cut-off index (COI) less than 1.0 was defined as negative, HBeAb and HBcAb antibody titer COI greater than 1.0 was defined as negative, and HBsAb antibody titer less than 10 mIU/ml was defined as negative. Meanwhile, we identified HBsAb titers ranging from 10 to 100 mIU/ml as weakly positive (*), and more than 100 mIU/ml was strongly positive (**). The HBV infection detection rate was defined as the percentage of HBcAb positive children among all children tested for HBcAb. The HBsAg positive rate was defined as the percentage of HBsAg-positive children among all children tested for HBsAg. The positive rate of HBV was defined as the percentage of HBV diagnosed patients in all HBV screened population during the same period.

### Statistical analysis

Hepatitis B infection rates in the overall populations and subgroups were evaluated by frequency, incidence and composition. The Cochran-Armitage test was used to analyze the changing trend of each subgroup across different years. R*C Chisq-Test and Ridit test were used to compare infection rates among subgroups. Statistical significance was determined using a bilateral *P* < 0.05. All statistical analyses were performed in SAS 9.2 and Graphd Prism 8.0.

## Results

### Characteristics of HBV serological markers

Here, we found and analysed five serological markers in all 115,029 participants. Of the total participants, 73,889 (64.24%) were males and 41,140 (35.76%) were females. The overall positive detection rates of HBsAg, HBsAb, HBeAg, HBeAb, and HBcAb in all participants were 0.13%, 79.09%, 0.17%, 2.81%, and 5.82%, respectively. The positivity rate of HBeAb and HBcAb in males was significantly lower than that in females (2.64% vs. 3.13%, 5.56% vs. 6.29%; *P* < 0.001). The positivity rates of HBsAg, HBsAb, and HBeAg in males and females were not statistically significant (*P* > 0.05) (Table 1).

**Table 1:** Detection characteristics of five HBV serological markers

Marker	Type	Total(N,%,95%CI)	Male(N,%,95%CI)	Female(N,%,95%CI)	χ^2^	*P*
HBsAg	(+)	153(0.13, 0.11-0.15)	103(0.14, 0.11-0.17)	50(0.12, 0.09-0.16)	0.63	0.43
	(-)	114,876(99.87, 99.85-99.89)	73,786(99.86, 99.83-99.89)	41,090(99.88, 99.84-99.91)		
HBsAb	(+)	90,978(79.09, 78.86-79.33)	58,542(79.23, 78.94-79.52)	32,436(78.84, 78.45-79.24)	2.39	0.12
	(-)	24,051(20.91, 20.67-21.14)	15,347(20.77, 20.48-21.06)	8704(21.16, 20.76-21.55)		
HBeAg	(+)	197(0.17, 0.15-0.20)	127(0.17, 0.14-0.20)	70(0.17, 0.13-0.21)	0.00	0.95
	(-)	114,832(99.83, 99.80-99.85)	73,762(99.83, 99.80-99.86)	41,070(99.83, 99.79-99.87)		
HBeAb	(+)	3237(2.81, 2.72-2.91)	1949(2.64, 2.52-2.75)	1288(3.13, 2.96-3.30)	23.49	<0.0001
	(-)	111,792(97.19, 97.09-97.28)	71,940(97.36, 97.25-97.48)	39,852(96.87, 96.70-97.04)		
HBcAb	(+)	6693(5.82, 5.68-5.95)	4106(5.56, 5.39-5.72)	2587(6.29, 6.05-6.52)	25.79	<0.001
	(-)	108,336(94.18, 94.05-94.32)	69,783(94.44, 94.28-94.61)	38,553(93.71, 93.48-93.95)		

### Distribution characteristics of HBV serologic positive markers

**Fig. 1:**
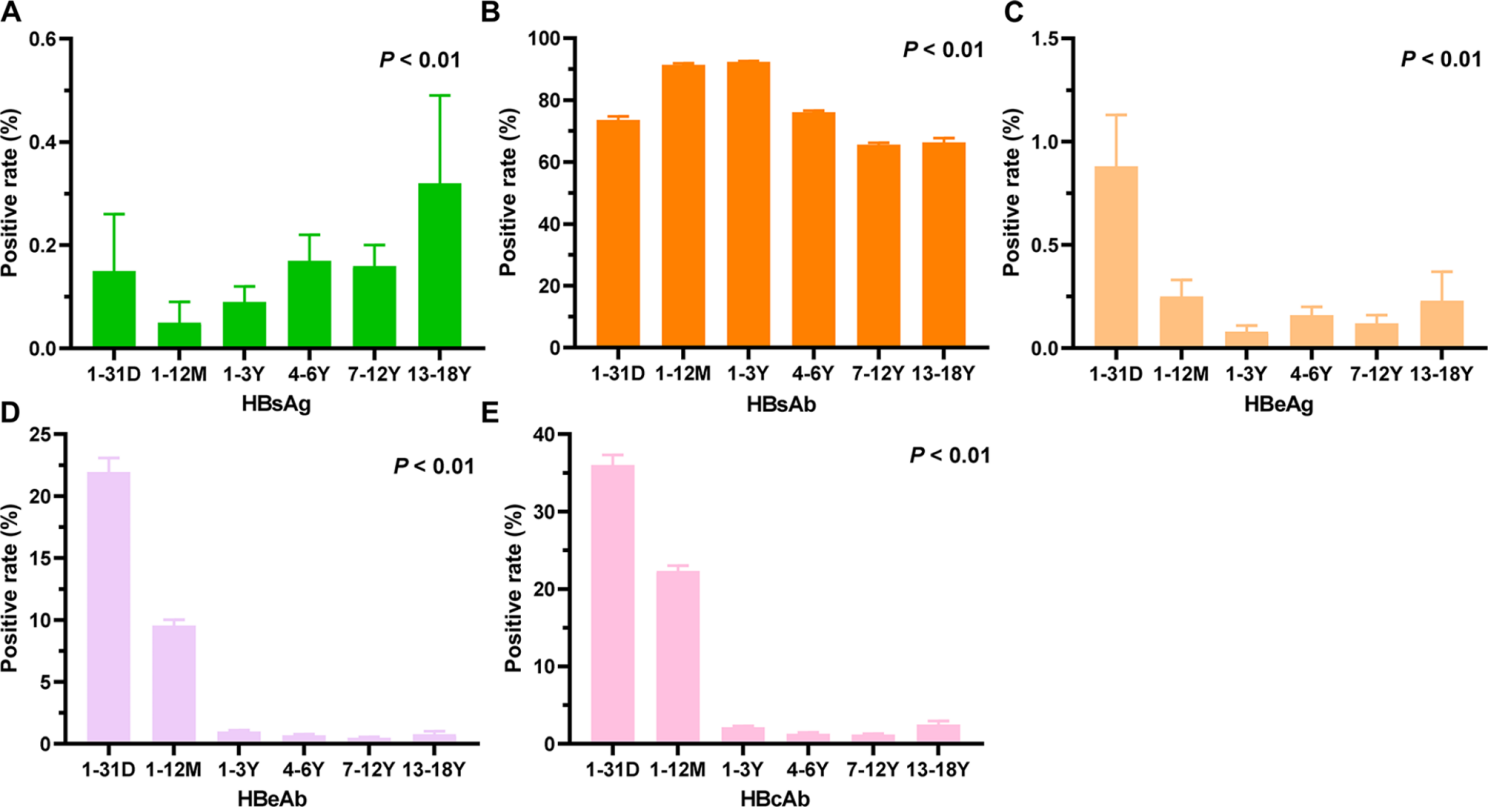
Percentage of positive HBV serologic markers in children of different ages. (**A**) HBsAg positive percentage among children in the six age groups. (**B**) HBsAb positive percentage among children in the six age groups. (**C**) HBeAg positive percentage among children in the six age groups. (**D**) HBeAb positive percentage among children in the six age groups. (**E**) HBcAb positive percentage among children in the six age groups

The analysis of age distribution showed that the positivity rates of the HBV serological markers in all age groups were statistically significant (*P* < 0.01). The positivity rate of HBsAg was higher in the neonatal period (1-31d) and lowest in the 1–12 m group, but then increased gradually with increasing age (Fig. 1A). The positivity rate of HBsAb increased gradually with increasing age, reaching a peak (91%) in 1 m-3y, then gradually decreasing with increasing age, stabilizing at 65% after 7y (Fig. 1B). The positivity rates of HBeAg, HBeAb and HBcAb showed similar trends, peaking in the neonatal period (1-31d) and then decreasing significantly with age. However, the positivity rate of HBeAg was lowest at 1-3y and gradually increased thereafter (Fig. 1C). The positivity rate of HBeAb and HBcAb was lowest at 7-12y, and then increased slightly (Fig. 1D, E). No significant association was found between the positivity rate and age (*P* > 0.05) (Supplementary Fig. 1 and Supplementary Table 1).

### Analysis of the serum HBsAb titre in children

**Fig. 2:**
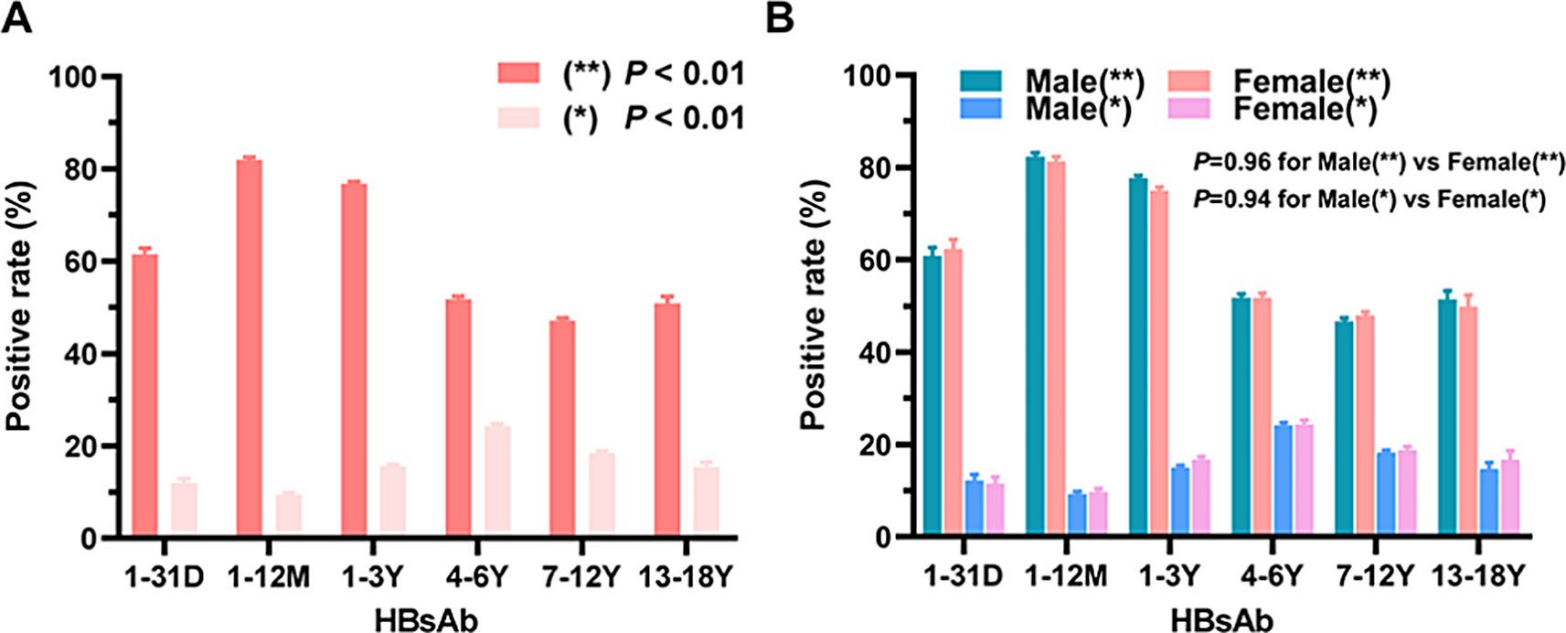
Percentage of positive HBsAb titter in children of different ages. (**A**) HBsAb (**) and (*) percentage among children in the six age groups. (**B**) HBsAb (**) and (*) percentage (%) among different sex children in the six age groups

High-titre HBsAb is considered to be the simplest and most effective way of protecting the body from HBV infection, therefore, further analysis of changes in HBsAb levels in children is of great value to judge the ability of children to resist HBV infection. Here, we classified HBsAb positive children into strongly positive (**) and weakly positive (*); the results showed that the positivity rates of the children that were strongly HBsAb positive gradually decreased and stabilized at 50% after peaking at 81.94% at 1 m-3y, and that nearly 15% of children were weakly HBsAb positive (Fig. 2A). No significant association was found between the positivity rate and sex (*P* > 0.05) (Fig. 2B).

### Serological pattern distribution of HBV markers

After infection with HBV, a series of regular changes in antigens and antibodies occur in serum, so the study of the combination pattern of HBV serologic markers is not only helpful in judging the immune status of the population against HBV, but also in assessing the treatment and prognosis of patients with HBV. As shown in Table 2, a total of 22 different HBV serological patterns was revealed. The top four serological combination patterns of normal HBV free were ①②③④⑤ all negative, ②, ②⑤, and ②④⑤ positive, accounting for 73.54%, 20.53%, 2.78%, and 2.60%, respectively. The top four serological combination patterns of children infected with HBV were ①③⑤, ②③⑤, ①②③⑤, and ①④⑤ positive, accounting for 0.08%, 0.06%, 0.02% and 0.02%, respectively. There were no significant differences in the serological patterns between males and females (*P* < 0.05). In Table 3, the distribution of the 22 serological patterns in different age groups show that the highest HBsAb positivity rate was in the 1–3 year age group, patients with HBV were mainly concentrated in the 4–12 year age group, and that children under 1 year of age presented mainly with the special combination pattern.

**Table 2:** Serological pattern distribution of the five HBV markers in sex groups

Patterns	Total(N=115,029)		Male(N=73,889)		Female(N=41,140)	
	N	%	N	%	N	%
**Negative**	23,614	20.53	15,064	20.39	8550	20.78
②	84,595	73.54	54,637	73.94	29,958	72.82
②⑤	3202	2.78	1989	2.69	1213	2.95
②④⑤	2993	2.60	1795	2.43	1198	2.91
⑤	177	0.15	117	0.16	60	0.15
④⑤	111	0.10	70	0.09	41	0.10
②④	93	0.08	57	0.08	36	0.09
①③⑤	89	0.08	56	0.08	33	0.08
②③⑤	67	0.06	43	0.06	24	0.06
①②③⑤	21	0.02	15	0.02	6	0.01
①④⑤	18	0.02	12	0.02	6	0.01
④	16	0.01	11	0.01	5	0.01
①③	9	0.01	8	0.01	1	0.00
①	7	0.01	4	0.01	3	0.01
③⑤	7	0.01	3	0.00	4	0.01
①②	2	0.00	2	0.00	0	0.00
①②④⑤	2	0.00	2	0.00	0	0.00
①③④⑤	2	0.00	1	0.00	1	0.00
①②③④⑤	1	0.00	1	0.00	0	0.00
①②⑤	1	0.00	1	0.00	0	0.00
①⑤	1	0.00	1	0.00	0	0.00
②③④⑤	1	0.00	0	0.00	1	0.00

**Table 3:** Serological pattern distribution of the five HBV markers in different age groups

Patterns	Total(N, %)	Age Groups(N, %)					
		1-31D	1-12 M	1-3Y	4-6Y	7-12Y	13-18Y
**Negative**	23,614(20.53)	1235(23.65)	1150(7.80)	2389(7.57)	6400(23.73)	10,996(34.16)	1444(33.23)
②	84,595(73.54)	2099(40.20)	10,284(69.79)	28,475(90.19)	20,169(74.79)	20,776(64.55)	2792(64.24)
②⑤	3202(2.78)	630(12.06)	1771(12.02)	368(1.17)	168(0.62)	203(0.63)	62(1.43)
②④⑤	2993(2.60)	1069(20.47)	1368(9.28)	274(0.87)	141(0.52)	113(0.35)	28(0.64)
⑤	177(0.15)	60(1.15)	86(0.58)	5(0.02)	4(0.01)	17(0.05)	5(0.12)
④⑤	111(0.10)	74(1.42)	29(0.20)	1(0)	0(0)	7(0.02)	0(0.00)
②④	93(0.08)	2(0.04)	10(0.07)	30(0.1)	35(0.13)	16(0.05)	0(0.00)
①③⑤	89(0.08)	0(0.00)	5(0.03)	16(0.05)	33(0.12)	26(0.08)	9(0.21)
②③⑤	67(0.06)	37(0.71)	30(0.20)	0(0)	0(0)	0(0.00)	0(0.00)
①②③⑤	21(0.02)	1(0.02)	2(0.01)	4(0.01)	7(0.03)	7(0.02)	0(0.00)
①④⑤	18(0.02)	1(0.02)	0(0.00)	3(0.01)	4(0.01)	7(0.02)	3(0.07)
④	16(0.01)	0(0.00)	0(0.00)	2(0.01)	5(0.02)	8(0.02)	1(0.02)
①③	9(0.01)	0(0.00)	0(0.00)	2(0.01)	2(0.01)	4(0.01)	1(0.02)
①	7(0.01)	4(0.08)	1(0.01)	0(0)	0(0)	2(0.01)	0(0.00)
③⑤	7(0.01)	7(0.13)	0(0.00)	0(0)	0(0)	0(0.00)	0(0.00)
①②	2(0.00)	2(0.04)	0(0.00)	0(0)	0(0)	0(0.00)	0(0.00)
①②④⑤	2(0.00)	0(0.00)	0(0.00)	0(0)	0(0)	1(0.00)	1(0.02)
①③④⑤	2(0.00)	0(0.00)	0(0.00)	2(0.01)	0(0)	0(0.00)	0(0.00)
①②③④⑤	1(0.00)	0(0.00)	0(0.00)	0(0)	0(0)	1(0.00)	0(0.00)
①②⑤	1(0.00)	0(0.00)	0(0.00)	0(0)	0(0)	1(0.00)	0(0.00)
①⑤	1(0.00)	0(0.00)	0(0.00)	0(0)	0(0)	1(0.00)	0(0.00)
②③④⑤	1(0.00)	1(0.02)	0(0.00)	0(0)	0(0)	0(0.00)	0(0.00)

### Characteristics of the HBV Infection detection rate

**Fig. 3:**
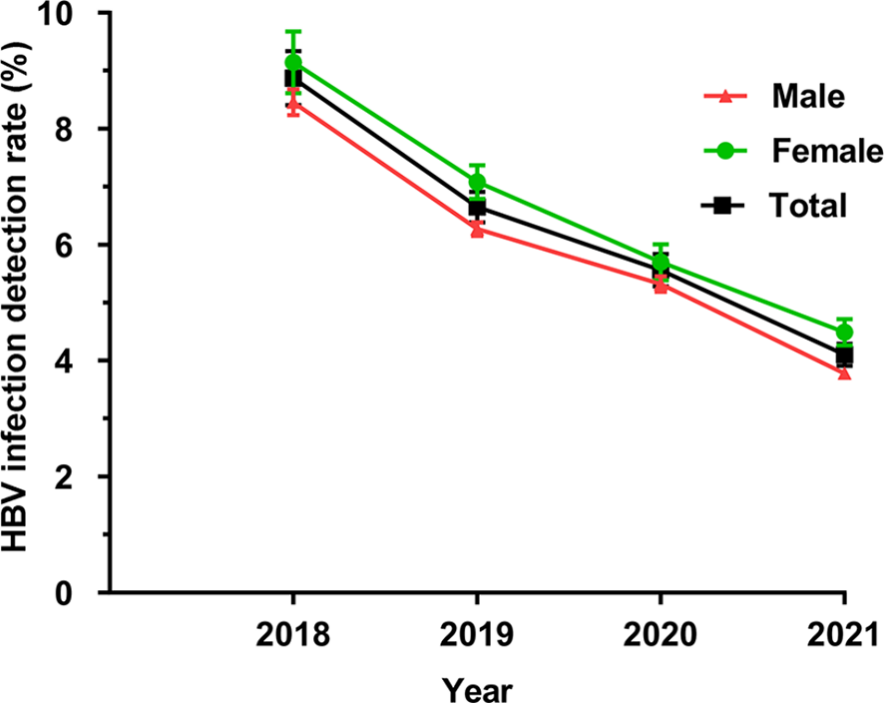
Characteristics of the HBV infection detection rate (%) in children from 2018 to 2021

HBV-infected people generally produce high titres of HBcAb, which persist for life, so the HBcAb positivity rate is a good way to assess the status of HBV infection in the region. In this study, 6693 children showed positive HBcAb, and the overall HBV infection detection rate was 5.82% [5.68–5.95]. The overall HBV infection detection rate in females was 6.29% [6.05, 6.35], which was higher than that in males at 5.56% [5.39, 5.59] (*P* < 0.01). The rate of HBV infection detection from 2018 to 2021 showed a decreasing trend year by year (*P* = 0.01), with the decline being most significant from 2018 to 2019 (Fig. 3). Comparing the differences in the HBV infection detection rate between males and females in different age groups, it was found that the rate in females in the 1 M-6Y age range was significantly higher than that in males (*P* < 0.05), and there were no statistically significant differences between the remaining age groups (*P* > 0.05) (Table 4). A comparison of the seasonal HBV infection detection rate from 2018 to 2021 showed significant differences between different years and seasons, it was highest in winter and spring but lowest in summer and autumn (*P* < 0.01) (Table 5).

**Table 4:** Characteristics of the total HBV infection prevalence rate(%) in different age and sex groups

Age Group	N	Overall(N,%,95%CI)	Male(N,%,95%CI)	Female(N,%,95%CI)	χ^2^	*P*
1-31D	5222	1880(36.00, 34.7-37.3)	1118(36.36, 34.66-38.06)	762(35.49, 33.47-37.52)	0.41	0.52
1-12 M	14,736	3291(22.33, 21.66-23.01)	2065(21.63, 20.81-22.46)	1226(23.62, 22.46-24.77)	7.63	0.01
1-3Y	31,571	673(2.13, 1.97-2.29)	411(1.96, 1.77-2.15)	262(2.47, 2.24-2.85)	8.94	<0.01
4-6Y	26,968	357(1.32, 1.19-1.46)	193(1.13, 0.97-1.29)	164(1.66, 1.41-1.91)	13.6	<0.01
7-12Y	32,186	384(1.19, 1.07-1.31)	249(1.23, 1.07-1.38)	135(1.14, 0.95-1.33)	0.51	0.47
13-18Y	4346	108(2.49, 2.02-2.95)	70(2.42, 1.86-2.99)	38(2.60, 1.79-3.42)	0.13	0.72

**Table 5:** Characteristics of the HBV infection prevalence rate in four seasons

Year	Spring(%,95%CI)	Summer(%,95%CI)	Autumn(%,95%CI)	Winter(%,95%CI)	χ^2^	*P*
2018	10.37(9.30-11.44)	6.35(5.68-7.02)	9.27(8.30-10.25)	11.49(10.27-12.72)	71.8	<0.01
2019	7.18(6.64-7.72)	5.09(4.72-5.46)	6.85(6.33-7.37)	9.61(8.78-10.44)	125.24	<0.01
2020	8.69(7.73-9.66)	3.59(3.24-3.95)	4.95(4.45-5.45)	8.61(7.82-9.40)	227.8	<0.01
2021	4.94(4.49-5.39)	2.76(2.50-3.02)	4.43(3.98-4.89)	5.58(5.04-6.11)	126.9	<0.01
Total	6.97(6.65-7.29)	4.08(3.89-4.26)	5.96(5.68-6.24)	8.14(7.75-8.52)	493.18	<0.01

### Characteristics of the overall prevalence of HBV

**Fig. 4:**
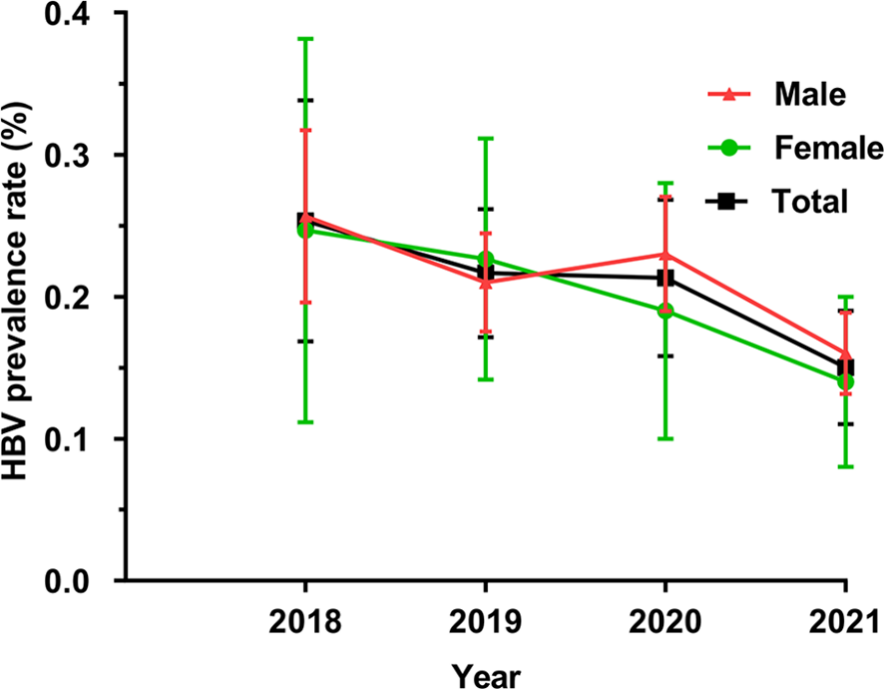
Characteristics of the overall prevalence of HBV in children from 2018 to 2021

In this study, 228 children were diagnosed with HBV. The overall proportion of HBV positive children in the 4 years was 0.2% [0.17, 0.22] and the overall rates in males and females were 0.2% [0.17, 0.23] and 0.19% [0.15, 0.23], respectively (*P* > 0.05). The overall prevalence of HBV decreased year by year from 2018 to 2021 (*P* = 0.01), with the most significant decrease occurring in 2020–2021 (Fig. 4). It was found that the rate in males was significantly higher than that in females in the 7–12 year age group (0.2% vs. 0.08%, *P* = 0.01), and that there was no statistically significant difference in other age groups (*P* > 0.05) (Table 6). In addition, the seasonal distribution of HBV in children showed that there was no significant statistical difference between the rates in the four seasons (Supplementary Table 1).

**Table 6:** Characteristics of the total prevalence of HBV among children in different age and sex groups

Age Group	Overall(N,%,95%CI)	Male(N,%,95%CI)	Female(N,%,95%CI)	χ^2^	*P*
1-31D	53(1.01, 0.74-1.29)	33(1.07, 0.71-1.44)	20(0.93, 0.53-1.34)	0.25	0.62
1-12 M	38(0.26, 0.18-0.34)	25(0.26, 0.16-0.36)	13(0.25, 0.11-0.39)	0.02	0.9
1-3Y	27(0.09, 0.05-0.12)	14(0.07, 0.03-0.10)	13(0.12, 0.06-0.19)	2.58	0.11
4-6Y	46(0.17, 0.12-0.22)	27(0.16, 0.10-0.22)	19(0.19, 0.11-0.28)	0.44	0.51
7-12Y	50(0.16, 0.11-0.20)	40(0.20, 0.14-0.26)	10(0.08, 0.03-0.14)	6.15	0.01
13-18Y	14(0.32, 0.15-0.49)	10(0.35, 0.13-0.56)	4(0.27, 0.01-0.54)	0.16	0.69

**Table 7:** Temporal and spatial variation in HBV types from 2018 to 2021

Variable	Total		Viral hepatitis type B(N,%,95%CI)			χ^2^	*P*
	N	n(%,95%CI)	Acute	Chronic	Unclassified		
Sex						0.54	0.91
Male	73,889	149(0.20, 0.17-0.23)	56(0.08, 0.06-0.10)	11(0.02, 0-0.03)	82(0.11, 0.09-0.13)		
Female	41,140	79(0.19, 0.15-0.23)	33(0.08, 0.05-0.11)	6(0.01, 0-0.03)	40(0.10, 0.07-0.13)		
Age						135.04	<0.0001
1-31D	5222	53(1.01, 0.74-1.29)	0	0	53(1.00, 0.74-1.29)		
1-12 M	14,736	38(0.26, 0.18-0.34)	5(0.03, 0-0.06)	0	33(0.22, 0.15-0.30)		
1-3Y	31,571	27(0.09, 0.05-0.12)	16(0.05, 0.03-0.08)	3(0.01, 0-0.02)	8(0.03, 0.01-0.04)		
4-6Y	26,968	46(0.17, 0.12-0.22)	33(0.12, 0.08-0.16)	4(0.01, 0-0.03)	9(0.03, 0.01-0.06)		
7-12Y	32,186	50(0.16, 0.11-0.20)	26(0.08, 0.05-0.11)	7(0.02, 0.01-0.04)	17(0.05, 0.03-0.08)		
13-18Y	4346	14(0.32, 0.15-0.49)	9(0.21, 0.07-0.34)	3(0.07, 0-0.15)	2(0.05, 0-0.11)		
Year						12.41	0.19
2018	14,250	36(0.25, 0.17-0.34)	16(0.11, 0.06-0.17)	3(0.02, 0-0.04)	17(0.11, 0.06-0.18)		
2019	36,064	78(0.22, 0.17-0.26)	32(0.09, 0.06-0.12)	4(0.01, 0-0.02)	42(0.12, 0.08-0.15)		
2020	25,671	55(0.21, 0.16-0.27)	16(0.06, 0.03-0.09)	4(0.02, 0-0.03)	35(0.14, 0.09-0.18)		
2021	39,044	59(0.15, 0.11-0.19)	25(0.06, 0.04-0.09)	6(0.02, 0-0.03)	28(0.07, 0.05-0.10)		

### Temporal and spatial variation in HBV types

**Fig. 5:**
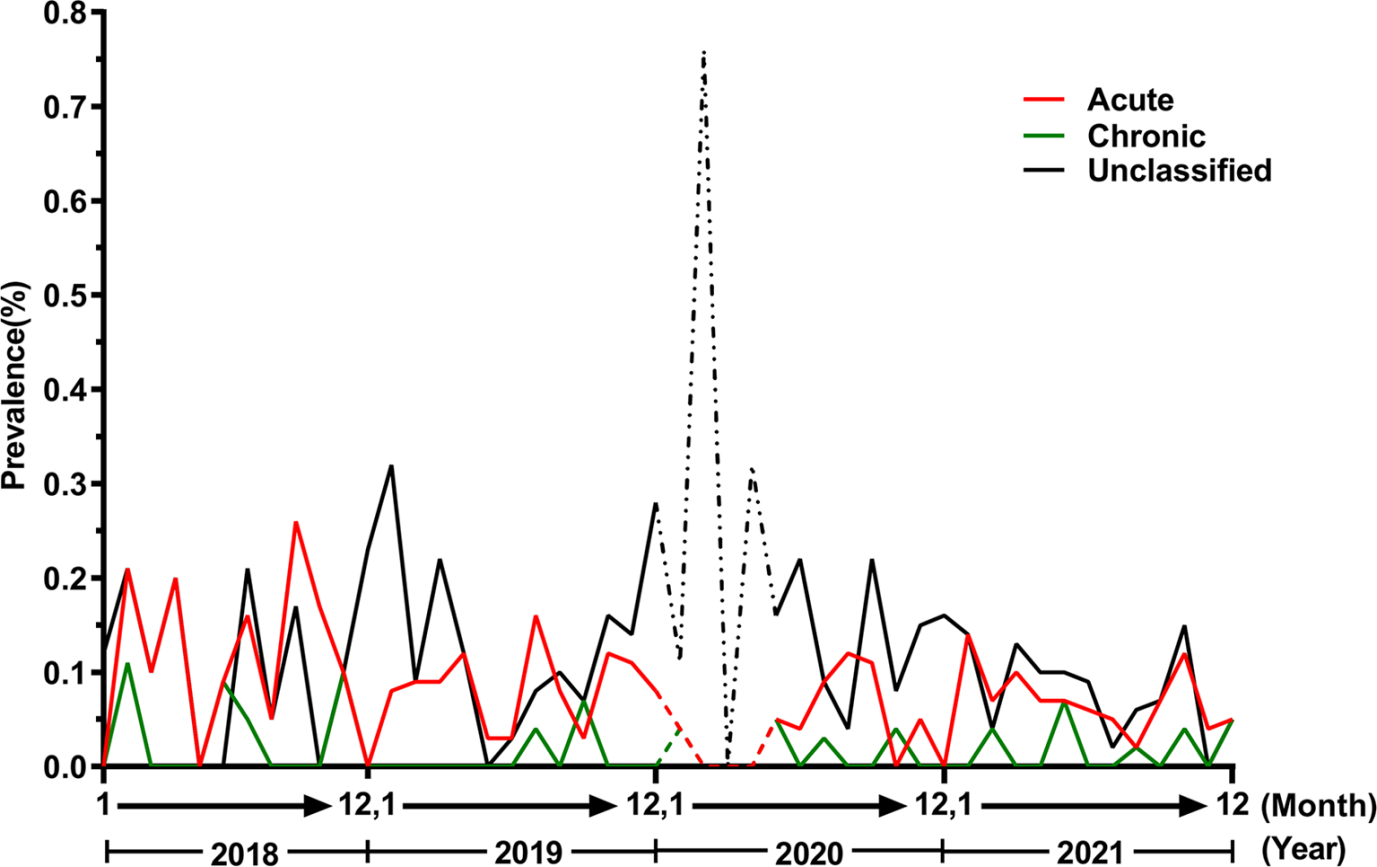
Temporal and spatial variation in HBV types from 2018 to 2021. Solid lines represent complete outpatient, emergency, and hospitalized child statistics, and the dashed line shows the missing data of outpatient children during the period from January to April 2020 due to the impact of COVID-19

Based on the outcomes of HBV serologic laboratory analysis, HBV infection can be categorized into three clinical types: acute, chronic, and unclassified HBV [17].The trends in different types from 2018 to 2021 showed that the incidence rate of acute infection was higher than that of other infection types most of the time, but the incidence of unclassified infection was higher after 2019 (Fig. 5). Comparing the changes in three types of HBV by sex and age showed no significant differences in the sexes (*P* > 0.05). However, there were significant differences among the age groups (*P* < 0.001). The infection types in infants (1-31d) and young children (1-12 m) were mainly unclassified HBV. From 1-12y, the incidence of acute and chronic HBV increased, particularly acute infection, while the unclassified type decreased significantly. In the 13-18y age group, acute HBV was the main type, but chronic and unclassified HBV also occurred frequently (Table 7).

## Discussion

HBV is still a major public health safety issue that has attracted much attention in China and the world, with vertical transmission from mother to child remaining an important route of HBV infection, accounting for nearly half of patients with HBV [18]. Therefore, it is very important and meaningful to study the prevalence of HBV infection in children. To better manage patients with HBV, the European Association for the Study of the Liver proposed a new nomenclature based on many biomarkers (Viral cccDNA, Hepatitis B core-related antigen [HBcrAg], and Circulating HBV RNA) for monitoring HBV infection in 2017, but these new biomarkers are still being evaluated and further studies are needed to provide clear evidence that the markers are superior to established HBV biomarkers, such as HBsAg and HBV DNA quantification, for clinical decision-making [19]. Therefore, the detection of HBV serological markers is still the best indicator of the status of HBV infection and replication in vivo, and for evaluation of the protective effect of the body against HBV [20].Herein, by conducting a systematic and detailed analysis of the prevalence and clinical characteristics of HBV infection in 115,029 children who have had testing of five serologic markers of HBV in our hospital in the past four years, we hope to provide important help for the next step, which is to improve HBV prevention and reduce the incidence of HBV infection.

According to the 2017 Global Hepatitis Report by WHO, about 3.5% (2.57 million) of the world’s population are chronically infected with HBV, and the HBsAg positivity rates are as high as 6.2% in the Western Pacific [21]. This study showed that the overall HBV infection detection rate in Wuhan was 5.82 [5.68, 5.95] %, the overall HBV prevalence rate accounted for only 0.2 [0.17, 0.22] %, and the total positive detection rate of HBsAg was as low as 0.13 [0.11, 0.15] %. Although Wuhan has yet to achieve the goal of reducing the prevalence rate of HBsAg in children to 0.1%, given the downward trend in positive HBsAg detection rates year by year and the optimization of pre-pregnancy care, we believe Wuhan will soon achieve it.

Next, we divided all the participants into six groups for a stratified analysis by age, and the results showed that HBsAg positive patients in this region were mainly infants and adolescents, similar to other regions [22]. Infections in infancy are mainly due to vertical transmission from mother-to-child, so further screening for HBV infection and taking the necessary treatment during pre-pregnancy and pregnancy will help reduce the transmission of HBV in infancy [18]. In addition, with the increased prevalence of HBV infection in adolescence, it is necessary to increase people’s awareness of HBV protection through more extensive scientific publicity, strengthening of HBV vaccination, and initiation of the corresponding early and timely treatment measures. This is because if this part of the population is not protected and treated, it will increase the circulating infection rate of the next generation of new-borns in the coming years [23].

HBsAb is an antibody produced by the body, stimulated by HBsAg, which has the effect of clearing the HBV to protect the body from infection [24]. Therefore, a positive HBsAb indicates that the body has strong immunity to the HBV. In this study, we found that HBsAb positivity increased with age from the neonatal period, which is closely related to our country’s first dose of HBV vaccine, which is administered within 24 h of birth, and regular subsequent vaccination with the second and third doses of HBV vaccine [25]. Meanwhile, we also found that, over time, the positivity rate of HBsAb gradually decreased from the age of 4 years, and basically stabilized at about 65% after the age of seven. In addition, about 20% of the children who were HBsAb positive had titres between 10 and 100 mIU/ml, which may be related to the vaccine protection time. Since the protective power of this titre against HBV decreases significantly, we hope that medical institutions or parents could evaluate the protective effect of the HBV vaccine at this age, and administer a booster dose of HBV vaccine if needed to improve their protection [26–28]. Concurrently, we found that more than 20% of children in the population were negative for HBsAb. For these children, whether to immediately boost HBV vaccination is still controversial. Some do not recommend immediate immunization of HBsAb-negative people with the HBV vaccine [29, 30]. Although HBsAb will gradually turn negative after vaccination with HBV vaccine, memory cells and long-term cellular immunity still exist to protect the vaccinated person from HBV infection. Another prospective cohort study has shown the need for an HBV vaccine in HBsAb-negative children, the pattern of HBV infection shifting from mother-to-child vertical transmission to horizontal transmission as children age, and that the main infected population will also change from new-borns to adults [31, 32]. In this study, we also found that adolescents aged 13–18 years have a significant increase in HBsAg positivity rates. However, many questions still exist, such as the age group that should be tested for HBsAb, or the intervals between tests; and for HBsAb-negative people, when to administer an HBV vaccine to boost immunity. These and many other issues need further study and solutions, as soon as possible. We believe that the establishment of comprehensive family-based prevention and control measures through continuous testing, immunization, prevention, and treatment is necessary to reduce the rate of HBV infection and improve protection against HBV.

The HBV serological markers are manifestations in the blood of the body’s immune response to different components of HBV, which can well reflect the current infection state of the body. In this study, we found a total of 22 different HBV serological patterns, including the four most common patterns in non-hepatitis B patients: ①②③④⑤all negative, ②, ②⑤, and ②④⑤ positive. The four most common patterns in patients with HBV were ①③⑤, ②③⑤, ①②③⑤, and ①④⑤ positive, accounting for 99.63% of the total number of people tested. The remaining 14 uncommon special serological patterns accounted for only 0.37%, and from the age distribution, we can see that these occurred mainly in children with HBV, particularly new-borns, which may be closely related to direct maternal infection. Concurrently, we also need to note that the emergence of these special serological patterns is also closely related to the following: early stage of atypical and subclinical infection or the antigen-antibody seroconversion stage [33, 34], early or latent stage of acute HBV infection [35]; different serum subtypes or S gene immune escape from infection with mutant strains, point mutations in the pre-core region of HBV, and so on. In conclusion, we should dynamically observe the changes in HBV serum markers and HBV DNA for these groups, and closely follow up to achieve targeted therapy for these special serological pattern patients. For this point, this study lacks corresponding data, and we will delve into this in future.

Acute and chronic HBV infection are easily diagnosed and treated to reduce the damage to the body. However, unclassified hepatitis B is not easy to diagnose, and can not only cause the spread of HBV, but also easily cause the occurrence of many chronic liver diseases [36]. In this study, we conducted a classification analysis of 228 children with confirmed HBV infection, and the results showed that the incidence of acute HBV infection was higher than that of chronic and unclassified infection in the vast majority of cases, but the incidence of unclassified HBV infection showed a significant upward trend after 2019. The incidence became gradually higher than that of acute and chronic HBV infection, but the cause is not clear at present. We hypothesised that it may be due to an increase in the activity of occult HBV in vivo, resulting in breakthrough infection, or due to the spread of HBV caused by infection. Then, because this survey is a single center retrospective study, there is a lack information about the examination and treatment of HBV before and after the mother’s pregnancy, the disease status of the child’s family, the status of HBV vaccination, the living environment. These aspects may not be well analysed and need to be studied more carefully and deeply in the future.

In conclusion, this study analysed the characteristics of HBV infection in children in Wuhan in detail, and found that although the overall HBV infection detection rate in Wuhan has decreased year by year, infants, young children under 1 year of age, and adolescents aged 13–18 years, still experienced a high incidence of HBV, and unclassified HBV increased significantly after 2019. HBV infection prevention education, pre-pregnancy HBV infection screening, virus blockade therapy, and HBV vaccination are still very necessary, and are of great guiding significance for the prevention and control of HBV infection in this region.

### Electronic supplementary material

Below is the link to the electronic supplementary material.

Supplementary Material 1

Supplementary Material 2

Supplementary Material 3

Supplementary Material 4

## Data Availability

The data that support the findings of this study are available on request from the corresponding author C Yao. The data are not publicly available due them containing information that could compromise research participant privacy.

## Abbreviations

HBV: Hepatitis B virus
HCC: Hepatocellular carcinoma
HBsAg: Hepatitis B surface antigen
HBsAb: Hepatitis B surface Antibody
HBeAg: Hepatitis B e Antigen
HBeAb: Hepatitis B e antibody
HBcAb: Hepatitis B core antigen
TBIL: Total bilirubin
DBIL: Direct bilirubin
IBIL: Indirect bilirubin
ALT: Alanine aminotransferase
AST: Aspartate aminotransferase
TP: Total Protein
ALB: albumin
GLB: Globulin
ALP: Alkaline phosphatase
γ-GT: γ-Glutamyl transpeptidase
ECLIA: Electrochemiluminescence immunoassay

## References

[1] Xu R, Hu P, Li Y, Tian A, Li J, Zhu C (2021). Advances in HBV Infection and replication systems in vitro. Virol J.

[2] Polaris Observatory C (2018). Global prevalence, treatment, and prevention of Hepatitis B virus Infection in 2016: a modelling study. Lancet Gastroenterol Hepatol.

[3] Suesstrunk J, Djongali FB (2017). Hepatitis B virus prevalence in rural areas in South-West Chad. Trop Doct.

[4] Norden C, Malham M, Nordly S, Grosen D, Kvistgaard H, Kjaer MS, Brix Christensen V (2020). Paediatric Hepatitis B and Hepatitis C virus Infections: an observational study of a Danish cohort. Acta Paediatr.

[5] Stinco M, Rubino C, Trapani S, Indolfi G (2021). Treatment of Hepatitis B virus Infection in children and adolescents. World J Gastroenterol.

[6] Paganelli M, Stephenne X, Sokal EM (2012). Chronic Hepatitis B in children and adolescents. J Hepatol.

[7] Sokal EM, Paganelli M, Wirth S, Socha P, Vajro P, Lacaille F, Kelly D, Mieli-Vergani G (2013). European Society of Pediatric Gastroenterology H, Nutrition: management of chronic Hepatitis B in childhood: ESPGHAN clinical practice guidelines: consensus of an expert panel on behalf of the European Society of Pediatric Gastroenterology, Hepatology and Nutrition. J Hepatol.

[8] Indolfi G, Easterbrook P, Dusheiko G, Siberry G, Chang MH, Thorne C, Bulterys M, Chan PL, El-Sayed MH, Giaquinto C (2019). Hepatitis B virus Infection in children and adolescents. Lancet Gastroenterol Hepatol.

[9] Wilkins T, Sams R, Carpenter M (2019). Hepatitis B: Screening, Prevention, diagnosis, and treatment. Am Family Phys.

[10] Yoda T, Katsuyama H (2021). Analysis of antibody-negative medical students after Hepatitis B vaccination in Japan. Hum Vaccines Immunotherapeutics.

[11] Cui F, Luo H, Wang F, Zheng H, Gong X, Chen Y, Wu Z, Miao N, Kane M, Hennessey K (2013). Evaluation of policies and practices to prevent mother to child transmission of Hepatitis B virus in China: results from China GAVI project final evaluation. Vaccine.

[12] Zou L, Ruan S, Zhang W (2015). On the sexual transmission dynamics of Hepatitis B virus in China. J Theor Biol.

[13] Liao X, Liang Z (2015). Strategy vaccination against Hepatitis B in China. Hum Vaccines Immunotherapeutics.

[14] Tordrup D, Hutin Y, Stenberg K, Lauer JA, Hutton DW, Toy M, Scott N, Bulterys M, Ball A, Hirnschall G (2019). Additional resource needs for viral hepatitis elimination through universal health coverage: projections in 67 low-income and middle-income countries, 2016-30. The Lancet Global Health.

[15] Chilaka VN, Konje JC (2021). Viral Hepatitis in pregnancy. Eur J Obstet Gynecol Reprod Biol.

[16] Diagnostic criteria for viral hepatitis B (WS299-2008.). http://www.nhc.gov.cn/wjw/s9491/200907/41983.shtml. 2008.

[17] Meng TT, Miao N, Wang FZ, Zheng H, Yin ZD, Liang XF, Zhang GM. Analysis on Hepatitis B cases reported from surveillance points in China, 2019. Zhonghua Liu Xing Bing Xue Za Zhi. 2021;42(9):1532–6. 10.3760/cma.j.cn112338-20210319-00233 34814580

[18] Veronese P, Dodi I, Esposito S, Indolfi G (2021). Prevention of vertical transmission of Hepatitis B virus Infection. World J Gastroenterol.

[19] European Association for the Study of the Liver (2017). Electronic address eee, European Association for the study of the L: EASL 2017 clinical practice guidelines on the management of Hepatitis B virus Infection. J Hepatol.

[20] Wang DD, Yi LZ, Wu LN, Yang ZQ, Hao HY, Shi XH, Wang B, Feng SY, Feng YL, Wang SP (2019). Relationship between maternal PBMC HBV cccDNA and HBV serological markers and its Effect on HBV Intrauterine Transmission. Biomed Environ Sci: BES.

[21] WHO. : Global hepatitis report. https://www.who.int/hepatitis/publications/global-hepatitis-report2017/en/, 2017, 4. 2017.

[22] Malik R, Hardikar W (2016). Hepatitis B and C in Children. Indian J Pediatr.

[23] Cheung KW, Lao TT (2020). Hepatitis B - Vertical transmission and the prevention of mother-to-child transmission. Best Pract Res Clin Obstet Gynecol.

[24] Fu X, Chen J, Chen H, Lin J, Xun Z, Li S, Liu C, Zeng Y, Chen T, Yang B (2017). Mutation in the S gene of Hepatitis B virus and anti-HBs subtype-nonspecificity contributed to the co-existence of HBsAg and anti-HBs in patients with chronic Hepatitis B virus Infection. J Med Virol.

[25] Zhang W, Ji Z, Wang L, Xiao D, Yan Y (2015). A meta-analysis of HBsAg-positive rate among general Chinese populations aged 1–59 years. Infect Dis.

[26] Poorolajal J, Mahmoodi M, Majdzadeh R, Nasseri-Moghaddam S, Haghdoost A, Fotouhi A (2010). Long-term protection provided by Hepatitis B vaccine and need for booster dose: a meta-analysis. Vaccine.

[27] Mastrodomenico M, Muselli M, Provvidenti L, Scatigna M, Bianchi S, Fabiani L (2021). Long-term immune protection against HBV: associated factors and determinants. Hum Vaccines Immunotherapeutics.

[28] Khan T, Jung IH, Khan A, Zaman G (2017). Classification and sensitivity analysis of the transmission dynamic of Hepatitis B. Theor Biol Med Model.

[29] Zhao H, Zhou YH (2018). Revaccination against Hepatitis B in late teenagers who received vaccination during infancy: yes or no?. Hum Vaccines Immunotherapeutics.

[30] Immunity ECGHB (2000). Are booster immunisations needed for lifelong Hepatitis B immunity? European Consensus Group on Hepatitis B immunity. Lancet.

[31] Wang Y, Chen T, Lu LL, Wang M, Wang D, Yao H, Fan C, Qi J, Zhang Y, Qu C (2017). Adolescent booster with Hepatitis B virus vaccines decreases HBV Infection in high-risk adults. Vaccine.

[32] Qiu Y, Ren JJ, Wu ZK, Shen LZ, Shan H, Dai XW, Li J, Liu Y, Ren W, Yao J (2020). Strategies for Hepatitis B booster vaccination among children: an 8-year prospective cohort study. Hum Vaccines Immunotherapeutics.

[33] Kannan P, Subramanian P, Maiyalagan T, Jiang Z (2019). Cobalt oxide porous nanocubes-based Electrochemical Immunobiosensing of Hepatitis B Virus DNA in blood serum and urine samples. Anal Chem.

[34] Shariati M (2021). Impedimetric Biosensor for Monitoring complementary DNA from Hepatitis B Virus based on gold nanocrystals. J Electrochem Soc.

[35] Cao X, Shang QH, Chi XL, Zhang W, Xiao HM, Sun MM, Chen G, An Y, Lv CL, Wang L (2020). Serum N-glycan markers for diagnosing liver fibrosis induced by Hepatitis B virus. World J Gastroenterol.

[36] Miao N, Zheng H, Sun X, Shen L, Wang F, Cui F, Yin Z, Zhang G, Wang F (2019). Enhanced sentinel surveillance for Hepatitis B Infection in 200 counties in China, 2013–2016. PLoS ONE.

