# Supplementary material for: Epidemiological and clinical profile of pediatric hepatitis B virus infections in Wuhan: a retrospective cohort study
Source: BMC Pediatr. 2023 Dec 16;23:636. doi: 10.1186/s12887-023-04460-w (PMC10724974; doi:10.1186/s12887-023-04460-w)
Supplement: Supplementary file 1 — Supplementary Material 1 [file 12887_2023_4460_MOESM1_ESM.docx]

**Supplementary Figure 1** The positive rate (%) of HBV serologic markers in different genders children varied with age. **A.** HBsAg positive rate (%) among different genders children in the six age groups. **B.** HBsAb positive rate (%) among different genders children in the six age groups. **C.** HBeAg positive rate (%) among different gender children in six age groups. **D.** HBeAb positive rate (%)among children in the six age groups. **E.** HBcAb positive rate (%) among different genders children in the six age groups.
