# Supplementary figures and images for: Epidemiological and clinical profile of pediatric hepatitis B virus infections in Wuhan: a retrospective cohort study
Source: BMC Pediatr. 2023 Dec 16;23:636. doi: 10.1186/s12887-023-04460-w (PMC10724974; doi:10.1186/s12887-023-04460-w)

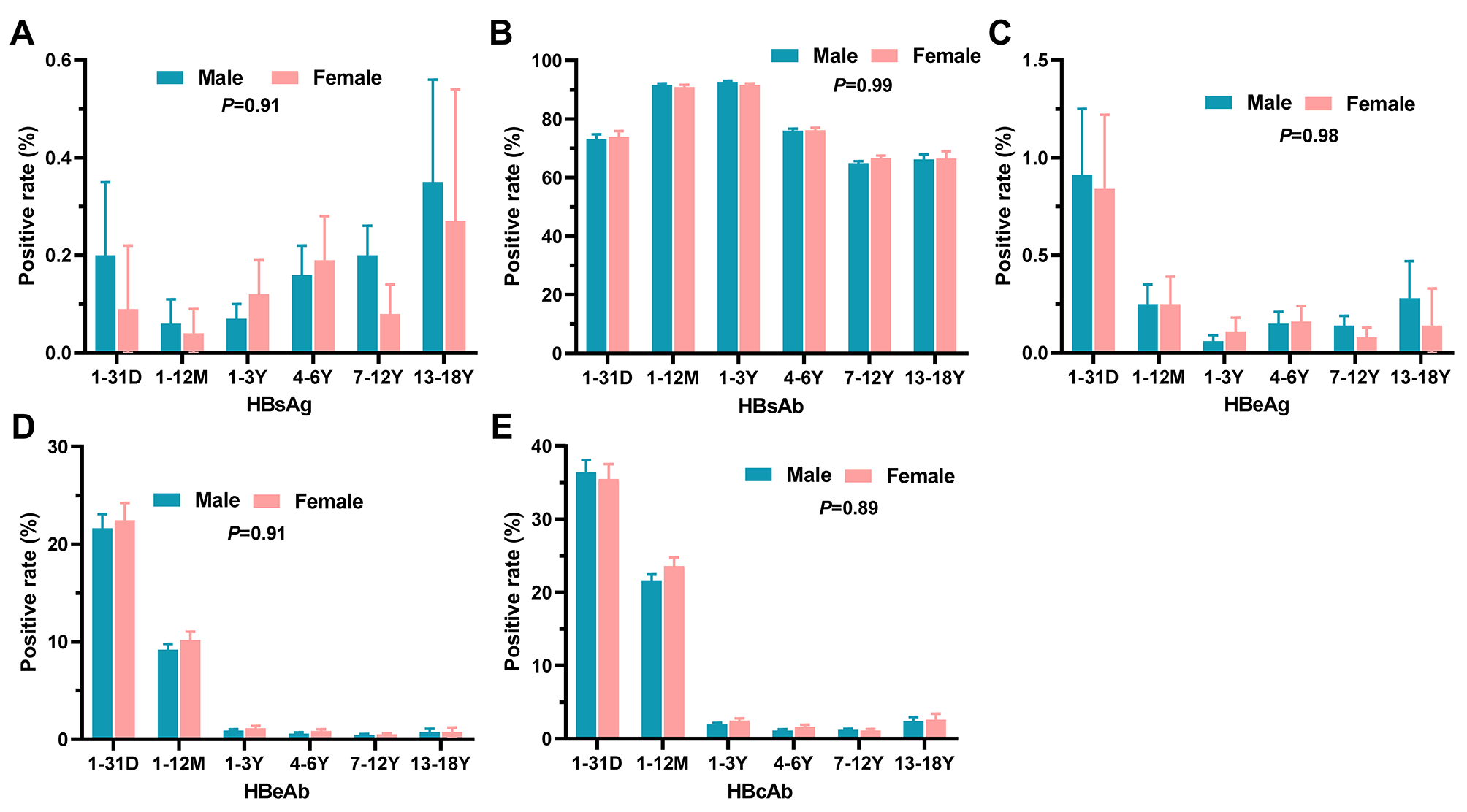

Supplement: Supplementary file 4 — Supplementary Material 4 [file 12887_2023_4460_MOESM4_ESM.png]
